# Uterine Metabolomics Reveals Protection of Taohong Siwu Decoction Against Abnormal Uterine Bleeding

**DOI:** 10.3389/fphar.2020.507113

**Published:** 2020-09-11

**Authors:** Yanyan Zhang, Chijing Zuo, Lan Han, Xiaochuang Liu, Weidong Chen, Jichen Wang, Shuangying Gui, Can Peng, Daiyin Peng

**Affiliations:** ^1^Department of Pharmacy, The First Affiliated Hospital of Anhui University of Chinese Medicine, Hefei, China; ^2^AnHui Province Key Laboratory of Chinese Medicinal Formula, Anhui University of Chinese Medicine, Hefei, China; ^3^Institute of Pharmaceutics, Anhui University of Chinese Medicine, Hefei, China; ^4^Anhui Province Key Laboratory of Pharmaceutical Preparation Technology and Application, Education Office of Anhui Province, Hefei, China

**Keywords:** abnormal uterine bleeding, Taohong Siwu decoction, metabolomics, UFLC-IT-TOF/MS, gas chromatography coupled with mass spectrometry

## Abstract

Incomplete abortion, a procedure for terminating pregnancy, will lead to abnormal uterine bleeding (AUB), infections, and even death. Taohong Siwu decoction (TSD) is a traditional Chinese medicine (TCM) formula, which has been developed to treat AUB for hundreds of years. However, the mechanism of the protective effect of TSD against AUB is not clear. We performed mass spectrometry (MS) of uterine samples to observe metabolic profile resulting from the treatment with TSD. An integrated gas chromatography-mass spectrometry and liquid chromatography-mass spectrometry based untargeted metabolomics approach combined with multivariate statistical analyses were used to investigate the metabolic profile of TSD against AUB. There was clear separation between pregnant and incomplete aborting rats as well as incomplete aborting and TSD administered rats. Based on random forest algorithm and receiver operator characteristic analysis, 12 biomarkers were optimized related to TSD administered. The effect of TSD on AUB are related to several pathways, such as AA metabolism, glyoxylate and dicarboxylate metabolism, alanine, aspartate, and glutamate metabolism. To our knowledge, this is the first uterine metabolomics study focusing on TSD on AUB and provide a new perspective for explaining the mechanism of TSD on AUB.

## Introduction

Since the early 2000s, medical abortion (MA) (mifepristone combined with misoprostol) has been accepted by more and more women who want to end the pregnancy (Singh et al., 2018). However, according to a new study by the Guttmacher Institute published in *The Lancet*, about 25 million unsafe abortions (45% of all abortions) occurred every year between 2010 and 2014 (Ganatra et al., 2017), which lead to incomplete abortion, abnormal uterine bleeding (AUB), vaginal, cervical, and uterine injury, infections, and even death (Ushie et al., 2018). It is of great significance to prevent and treat the complications caused by unsafe abortion.

Medical abortion is related to metabolism. It is reported that there is a metabolic dysfunction in idiopathic recurrent spontaneous miscarriage (Banerjee et al., 2014). ^1^H nuclear magnetic resonance (^1^H-NMR) metabolomics revealed the metabolic disturbance of amino acids (Wu et al., 2018) metabolism in aborting patients. Various metabolic abnormalities such as lipid and amino acid are considered to be major contributory factors to abortion (Gong, 2017). Consequently, a high probability that metabolites in women with history of MA will be differently expressed as compared to proven fertile women. Metabolomics has become a helpful platform for the identification of low-molecular weight metabolites in the biological system during a specific time (Nicholson et al., 2002). It is used to reflect the physiological effects of diseases (Wang et al., 2016) and clarify the role of traditional Chinese medicine formula on the treatment of diseases (Guo et al., 2016). Uterus can be sampled for metabolomic analysis to investigate specific information and provide a metabolic change that occurred within the tissue (Subramani et al., 2016). Though several metabolites may be secreted into the blood stream, mechanistic study associated with diseases originating from the affected uterus is expected to be more reliable compared with the blood samples.

Liquid chromatography coupled with mass spectrometry (LC/MS) and gas chromatography coupled with mass spectrometry (GC/MS) are the main techniques used for metabolomics (Alseekh and Fernie, 2018). Electron impact ionization (EI) of GC/MS is hard ionization, however, it is only suitable for substances with low boiling point and better thermostability. Polar metabolites are derivitized in order to render them volatile to separate. Electrospray ionization (ESI) is soft ionization, suitable for high boiling point and thermally unstable substances (Dunn et al., 2011; Gika et al., 2014). Consequently, the good complementarity of GC/MS and LC/MS facilitates a more comprehensive detection of the profile of endogenous metabolites.

Taohong Siwu Decoction (TSD), a traditional Chinese medicine formula, has been developed to treat AUB for hundreds of years. TSD was first recorded in *Yizong Jinjian* by Wu Qian in Qing Dynasty of China and has been widely used in clinics (Wu, 1997; Pan, 2014). TSD reduces lysophosphatidic acid (Li et al., 2013) and amino acid (Li et al., 2016) levels in AUB model rats, which indicated that the effect of TSD on AUB may relate to the lipid and amino acid pathways. This research intends to apply LC/MS, GC/MS metabolomics to explore the protective effect of TSD against AUB.

## Materials and Methods

### Chemicals and Reagents

N-methyl-N-trimethylsilyl trifluoroacetamide (MSTFA) and methoxyamine (MOX) were purchased from Sigma-Aldrich (St. Louis, MO, USA). Methanol, acetonitrile (HPLC grade) were obtained from Merck (Germany). Formic acid was obtained from Nanjing Chemical Reagent (Nanjing, China). Distilled water was filtered through a Milli-Q system from EMD Millipore Corporation (Billerica, MA, USA).

TSD is composed of Persicae Semen (batch number: 1709257), Carthami Flos (batch number: 1709295), Rehmanniae Radix (batch number: 1709293), Angelica Sinensis (batch number: 1709273), Paeoniae Radix Alba (batch number: 1709180352), Chuanxiong Rhizoma (batch number: 1709301) at the weight ratio of 3:2:4:3:3:2. Each herb which was purchased from the Bozhou Huqiao Pharmaceutical Industry Limited Company (Bozhou, China, Drug GMP certificate: AH20160273. Drug Manufacturing Certificate: Wan20160095), was identified by Professor Dequn Wang in the School of Pharmacy, Anhui University of Chinese Medicine. The detailed information of six plants was as follows:

1. Persicae Semen [*Prunus persica* (L.) Batsch, Rosaceae], which promotes blood circulation and removes blood stasis.
2. Carthami Flos (*Carthamus tinctorius* L., Compositae), which activates blood to promote menstruation and scatters stasis to alleviate pain.
3. Rehmanniae Radix (*Rehmannia glutinosa* Libosch., Scrophulariaceae), which enriches the blood and nourishes *Yin*.
4. Angelica Sinensis Radix [*Angelica sinensis* (Oliv.) Diels, Umbelliferae], which supplements the blood to activate blood circulation and adjusts menstruation for the pain.
5. Paeoniae Radix Alba (*Paeonia lactiflora* Pall., Ranunculaceae), which nourished the blood and regulates menstruation.
6. Chuanxiong Rhizoma (*Ligusticum chuanxiong* Hort., Umbelliferae), which promotes the circulation of blood and *Qi*.

### Preparation of TSD

TSD was prepared according to our previous report (Zhang et al., 2018). Briefly, six herbs were mixed at the weight ratio and extracted twice in ten and eight times (v/w) of 75% ethanol for 2 h, respectively. After filtering with eight layers carbasus, the decocted, evaporated, and concentrated decoction was suspended at the concentration of 2.4 g/ml. The decoction was lyophilized into powder and preserved at 4°C for subsequent use. The chemical components and the content of TSD are shown in **Supplementary Figure S1** and **Table S1**.

### Animal Experiments and Sample Collection

All animal experiments were performed according to the institutional guidelines for the care and use of laboratory animals and were approved by the Ethical Committee of Anhui Medical University (License Number: LLSC 20160336). Female Sprague-Dawley rats (240 ± 20 g body weight) and male rats (280 ± 20 g body weight) were obtained from the Laboratory Animal Centre of Anhui Medical University [License No. SCXK (Jin) 2016-0006] and acclimatized for 1 week prior to experiments with free access to food and water. The animals housed in a light and temperature controlled room (12/12 light/dark, 23–25 centigrade, 45–55% humidity).

The incomplete aborting model was established as previously described (Zhang et al., 2018). Rats were randomly divided into four groups, which included the pregnant (P), medical aborting (M), TSD (T), Yimucao Granule (Y) group. The cotton balls from vaginas were collected and stored at −20°C for the measurement of the volume of uterine bleeding. From day 8 of gestation, the rats in P and M group were treated with 1 ml per 100 g body weight distilled water each day for 7 days, the rats in group T were treated with TSD (9 g/kg), and the rats in group Y were treated with Yimucao Granule (4.5 g/kg). On day 14 of gestation, all rats were anesthetized with chloral hydrate and uteri were removed. The sinistro-uteri were stored at −80 centigrade; the dextro-uteri were fixed in 4% paraformaldehyde for 24–48 h at 4°C for pathologic evaluation.

### Determination of Uterine Bleeding Quantity

The uterine bleeding quantity was measured by the method of Alkaline Hematin Photometric as was our previously described (Zhang et al., 2018).

### Histopathological Examination

The dextro-uteri which were fixed in paraformaldehyde, and embedded in paraffin. Next, tissue sections were cut off with 4 µm and mounted on glass slides, then retained with hematoxylin-eosin (HE). Finally, the slides were sealed with neutral gum and the histopathological examination was evaluated using a microscope.

### Uteri Samples Pretreatment

Each sinistro-uterus tissue was measured precisely and added to precooled methanol (1:10, W/V, containing heptadecanoic acid, 5 μg/ml). Then each uterus was homogenized with tissue homogenizer (Bioprep-24 homogenizer, China). After centrifugation (14,000 *g*, 10 min, 4°C) for two times, the supernatant was transferred for metabolomic analysis (Yao et al., 2017).

### GC/MS Spectral Acquisition of Uteri Samples and Data Pretreatment

All the uterine samples were out of order. The supernatant (80 μl) was transferred into a glass vial, then oximated with 25 μ; of MOX hydrochloride (10 mg/ml) in pyridine at 1,200 rpm for 90 min at 37°C. The mixture was vacuum dried at 50°C for 2 h (Labconco CentriVap^®^, Kansas, MO, USA) and the residue were silylated with 120 μl of MSTFA, then incubated at 37°C for 2 h at 1,200 rpm. Supernatant was separated for GC/MS analysis.

The GC/MS analysis was operated using GCMS-QP2010 model (Shimadzu Co., Kyoto, Japan) equipped with a fused silica capillary column [Rtx-5MS; 30 m × 0.25 mm (inner diameter), film thickness: 0.25 μm; Restex]. The carrier gas was helium at a flow rate of 1 ml/min. The injector, transfer line, and ion source temperatures were carried out at 250, 250, and 200°C, respectively. The mass spectrometer was performed in electron impact mode (70 eV) at full scan mode from 45 to 600 (*m/z*) with a scan time of 0.2 s. The oven temperature was maintained at 70°C for 2 min, then increased to 320°C at a rate of 10°C/min and lasted for 2 min at 320°C. The run time was 29 min in total. Each sample (1 μl) was injected into GC/MS in split mode (50/L, v/v). The compounds were identified by comparing the mass spectra and retention time with those available in National Institute of Standards and Technology (NIST 5) using the similarity index (SI > 85) (Dai et al., 2016).

### UFLC-IT-TOF/MS Spectral Acquisition of Uteri Samples and Data Pretreatment

The UFLC-IT-TOF/MS analysis was performed using a Shimadzu Prominence series UFLC system *via* electrospray ionization (ESI) source. Chromatographic method was achieved adopting a Phenomenex Kinetex C18 column (100 × 2.1 mm, 2.6 μm; Phenomenex, Torrance, CA, USA) as a stationary phase and 0.1% formic acid (A)/acetonitrile (B) as a mobile phase with gradient elution at a constant flow rate of 0.4 ml/min. The elution order was: linear gradient from 5% B to 95% B in 20 min and maintained with 95% B in 3 min, then returned to 5% B for 7 min. The column temperature was carried out at 40°C. An aliquot of the supernatant (40 μl) was transferred into autosampler vial for analysis. Each sample (5 μl) was injected into UFLC-IT-TOF/MS for analysis and the sample cabinet temperature was 4°C (Dai et al., 2016).

The MS parameters were as follows: mass range was scanned from 100 to 1,000 mass-to-charge ratio (*m/z*) adopting an accumulation time of 20 ms per spectrum. The interface voltage was 4.5 kV for positive mode and −3.5 kV for negative mode. The detector voltage of the TOF analyzer was 1.65 kV. The heat block temperature and curved desorption line (CDL) were both 200°C. Nitrogen was used as the atomizer and dry gas, at a flow rate of 1.5 and 10 L/min, respectively. Argon was used as the colliding gas and the collision energy was set at 15, 25, 35, 45, 60, 80, or 100 eV in the tandem MS/MS mode, respectively. The spectral mass accuracy of the instrument was calibrated to an error of less than 5 ppm, using an external standard of sodium trifluoroacetate (STFA) solution. LCMS Solution software (version 3.0, Shimadzu) was used for instrument control, data acquisition, and data processing, including a formula predictor for predicting chemical formulas.

A multistep program was applied to identify the structure of the metabolites. The first step is the annotation of ions from the same metabolite. The second step is to use Formula Predictor to predict metabolites formula by comparing the theoretical, observed *m/z* value results and isotopic patterns. The third step is database and literature retrieval. Differential markers were compared with the *m/z*, formula and the MS/MS fragmentation information presented by Human Metabolome Database (HMDB; http://www.hmdb.ca), the online Metlin database (http://metlin.scripps.edu), and LIPID MAPS (http://www.lipidmaps.org).

### Data Pre-Processing

Chromatograms, obtained from GC/MS and LC/MS analysis, were processed for peak deconvolution and alignment, adopting the Profiling Solution version 1.1 (Shimadzu, Kyoto, Japan). The primary parameters were set as follows: ion m/z tolerance (500 mDa for GC/MS and 25 mDa for LC/MS), ion retention time tolerance (0.03 min for GC/MS and 0.5 min for LC/MS), width (5 s), slope (2,000 min^-1^), and ion intensity threshold (5,000 counts). Other parameters were set to default values. A matrix was generated and exported to an Excel table, which containing matched peaks with retention time, m/z value, and corresponding intensities. Solvent blanks were injected randomly to check the mass and exclude some sources of contamination, such as reagent impurities, contamination originating from sample preparation and instrument. We applied the so-called 80% rule to retain those metabolites detectable which were more than 80% subjects in at least one group in order to minimize the influence of the missing values. The exported matrix was further processed by removing the variables with relative standard deviation (RSD) higher than 30% in quality control (QC) samples. In the data table, the missing values were replaced with a half of the minimum value in the data set, then the total area normalization of each sample was performed (Chen et al., 2017).

### Statistical Analysis

Pre-processed data were imported to SIMCA-P version 13.0 (Umetrics, Sweden) to conduct multivariate statistical data analysis, for example, principle component analysis (PCA) and orthogonal partial least squares-discriminate analysis (OPLS-DA), with pareto scaling. We used logarithmic transformation to stabilize the variance across the intensity range (Rocke and Durbin, 2003). The PCA was used to observe grouping trends and outliers of the samples, in addition, the OPLS-DA was conducted for the selection of potential biomarkers based on variable importance (VIP value, VIP > 1) (Wiklund et al., 2008). Moreover, fold-change (FC, FC > 2 or FC < 0.5) and unpaired Mann-Whitney tests with Benjamini-Hochberg false discovery rate (FDR, FDR < 0.05) correction (Broadhurst and Kell, 2006) were used to select significant metabolites and reduce the probability of Type I errors (i.e. false positives). Spearman rank correlation analysis was performed to describe whether there was a close correlation. The higher the value, the stronger the correlation. The variables which meet the above conditions simultaneously were considered to have significant differences and conducted to identify structural and potential marker.

Classification performance of biomarkers was evaluated by an online receiver operator characteristic (ROC) curve tester (Xia et al., 2013) based on the Random Forest algorithm. The measure of classification performance was the area under the ROC curve (AUC), which values can be interpreted as an excellent classification performance ranging from 0.9 to 1.0.

Statistical differences in metabolites and the volume and duration of bleeding data among the three groups were performed by the nonparametric Kruskal-Wallis *H* test using SPSS 23.0 (IBM, Chicago, USA). Metabolites heatmap was drawn using MetaboAnalyst and other data were graphed using GraphPad Prism v5.01 software (GraphPad Software Inc, San Diego, CA, USA).

## Results

### Animal Models

**Figure 1:**
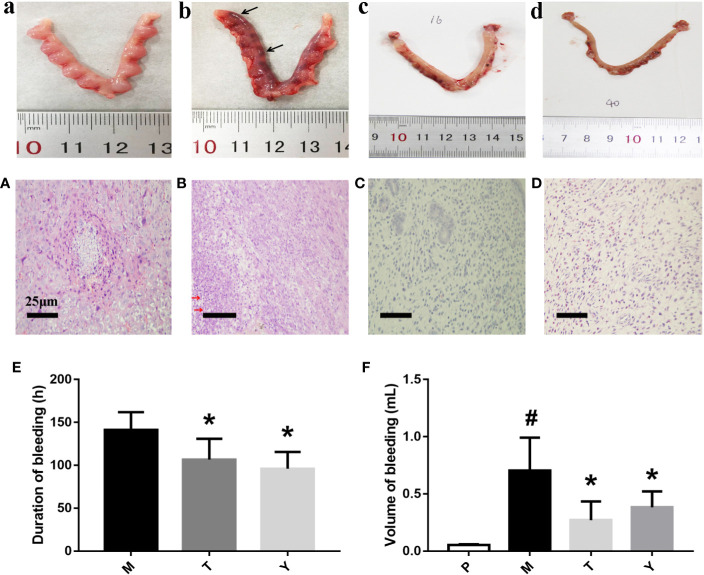
Macroscopic (a–d) and Pathological **(A–D)** observation of uteri tissue. Samples were performed with HE as described in Methods. a, A: group P; b, B: group M; c, C: group T. d, D: group Y. Black arrows indicate dark red residue, red arrows indicate necrotic decidual cells. Duration **(E)** and volume **(F)** of uterine bleeding. The duration of uterine bleeding was recorded from the beginning of uterine bleeding after the administration of mifepristone to the cessation of bleeding completely. The unit of duration was measured in hours. Adopting the method of Alkaline Hematin Photometric to measure the volume of uterine bleeding in different groups. The unit of volume is in ml. ^#^P < 0.05 *vs* group P. *P < 0.05 *vs* group M.

In our research, rats had a significant aggravation and prolongation of uterine bleeding in the medical aborting group (p < 0.05). Interestingly, rats in T and Y group dramatically reduced the volume of uterine bleeding and the duration of hemorrhage (**Figures 1E, F**). From macroscopic inspection, the dark red residue (black arrows) in drug treatment is less than medical aborting group (**Figures 1A–D**). From microscopic inspection, there was a large number of necrotic decidual cells (red arrows) in medical aborting group. After drug treatment, necrotic cells were less than medical aborting group (**Figures 1A–D**).

### Data Quality Assurance

All samples were randomized before analysis in order to avoid systematic variation from instrument-based analysis. QC samples, which monitored the robustness of sample preparation as well as the stability of instrument analysis (**Figure S2**), was tight clustered in the PCA scores (**Figure S3**) in GC/MS, UFLC-IT-TOF/MS (ESI+), and UFLC-IT-TOF/MS (ESI-). These results indicated a good reproducibility of the method of the analysis (Sánchez-Illana et al., 2018).

### Metabolic Profiling of Uterine Tissue Samples and Multiple Statistic Analysis

**Figure 2:**
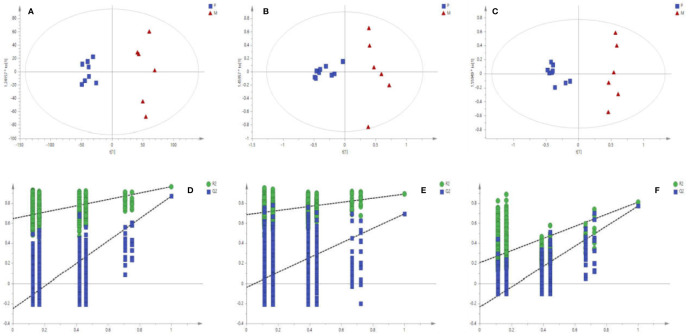
Score plots **(A–C)** of OPLS-DA Validation plots **(D–F)** obtained from 999 random permutation tests results between P (square) and M (triangle) in uterine tissues; A, D: GC/MS; B, E: UFLC-IT-TOF/MS (ESI+); C, F: UFLC-IT-TOF/MS (ESI-).

**Figure 3:**
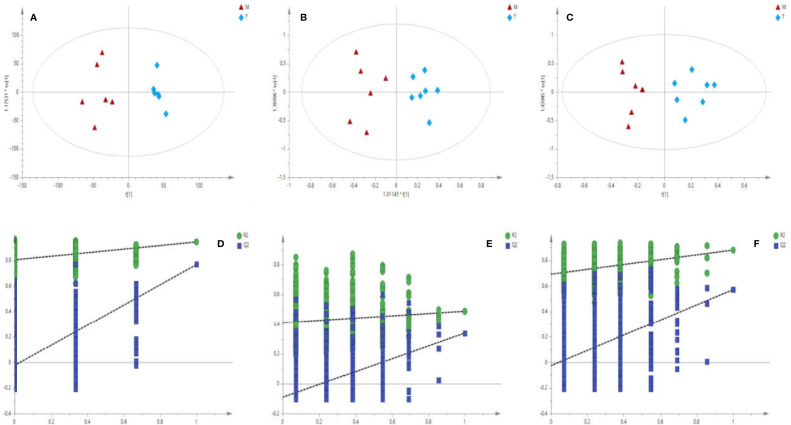
Score plots **(A–C)** of OPLS-DA Validation plots **(D–F)** obtained from 999 random permutation tests results between M (triangle) and T (rhombus) in uterine tissues; a, d: GC/MS; b, e: UFLC-IT-TOF/MS (ESI+); c, f: UFLC-IT-TOF/MS (ESI-).

The matrix of each sample peak intensities was from the original GC/MS, UFLC-IT-TOF/MS (ESI-), and UFLC-IT-TOF/MS (ESI+), respectively. As illustrated in **Figures S2 A–C**, typical total ion current chromatograms (TICs) of uterine tissues generated by GC/MS (A), UFLC-IT-TOF/MS (ESI+) (B), and UFLC-IT-TOF/MS (ESI-) (C). Classification trends among the three groups were obtained adopting PCA score plots (**Figure S3**) and OPLS-DA (**Figure S4**). In order to improve regional differences between groups and improve the effectiveness and resolution of the model, OPLS-DA analysis of P *vs* M (PM) (**Figures 2A–C**) and M *vs* T (MT) (**Figures 3A–C**) were conducted.

Based on the above data, the 999 random permutation test results in OPLS-DA models demonstrated clear separation in PM and MT rats with satisfactory discriminating ability (**Figures 2D–F** and **3D–F**). The seven-round internal cross-validation was used to validate the OPLS-DA models. The quality of the model was described using the goodness-of-fit parameter R2X, R2Y, and the predictive ability parameter Q2. The statistical model is generally considered effective and no overfitting when the Q2-intercept value is negative and the R2-value is lower than the original R2-value (Pasikanti et al., 2013; Triba et al., 2015; Zhang et al., 2017). An OPLS-DA model and the partial least squares-discriminant analysis (PLS-DA) model have the same total number of components. Analysis of variance testing of cross-validated predictive residuals (CV-ANOVA) tests were operated to determine significant differences among groups in the OPLS-DA models (Farshidfar et al., 2018). It was normally considered that *p* value lower than 0.05 as a significant model (Leruez et al., 2018). The PCA and the OPLS-DA models were shown in **Table S2**.

### Identification of Potential Biomarkers

**Figure 4:**
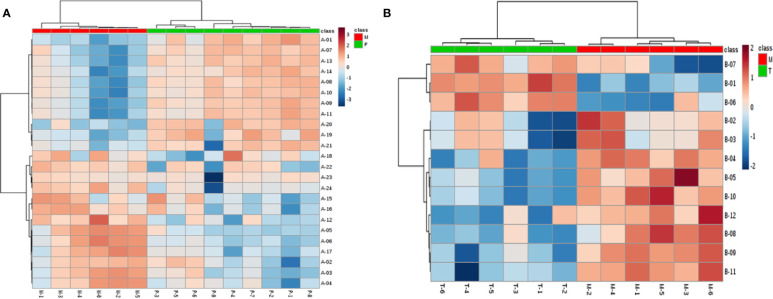
Biomarkers’ heatmap related to medical abortion **(A)** and intervention of TSD **(B)** in uterine tissue. Red area: an increase of up to twofolds; blue area: a reduction of up to twofolds.

To seek potential metabolites associated with PM and MT, we carefully selected a number of metabolites that contributed to their predictions. First, the VIP value for each model was considered. The VIP value greater than 1 was selected. The higher VIP score, the more reliable separation among the groups. Second, it was regarded as significant variable if the *p* value was less than 0.05. Structural identification was conducted according to the variables that simultaneously satisfy VIP > 1 and *p* < 0.05. There are 376, 292 differential variables associated with PM by GC and LC analysis. There are 328, 124 differential variables associated with MT by GC and LC analysis, respectively. After identification, 48 potential biomarkers were obtained associated with PM (GC: **Table S3**, LC: **Table S4**) and 39 potential biomarkers were obtained associated with MT (GC: **Table S5**, LC: **Table S6**). Fold change (FC) of each metabolite was calculated using the ratio of the two groups. Spearman correlation coefficient, unpaired Mann-Whitney tests with Benjamini-Hochberg FDR correction were performed as the final testing procedure and variables with significant differences (FDR < 0.05) were accepted. At last, the AUC was calculated. Above these metabolites, 24 metabolites related to PM and 12 metabolites related to MT were summarized in **Tables 1** and **2**. Metabolites related to PM included fatty acid (6) Cephalin (6), Lecithin (6), Energy (2), Carnitine (1), Bile Acid (1), Cholic Acid (1), Amino Acid (1). Metabolites related to MT included fatty acid (6), Amino Acid (4), Energy (1), Nucleotide (1). Heatmap of 24 or 12 differential metabolites was observed in **Figures 4A, B**. Changes of the contents of biomarkers in uterine tissue were demonstrated in **Figure S5**. Biomarkers ROC curves associated with PM and MT in uterine tissue were shown in **Figure S6**, **Figure S7**, respectively.

**Table 1:** Biomarkers associated with PM in uterine tissue.

	No	Metabolites	*P*-value	r	Adjusted *p*-value	AUC	Fold-change (M/P)(M/P)	FDR
Carnitine	A01	L-Acetylcarnitine	2.00E-03	−0.722a	2.00E-03	0.98	0.38↓	7.71E-03
Cephalin	A02	PE (P-16:0e/0:0)	5.00E-03	0.763a	1.00E-03	0.94	3.07↑	1.67E-02
Cephalin	A03	LysoPE (16:0)	5.00E-03	−0.582a	1.00E-03	0.98	0.49↓	3.44E-02
Cephalin	A06	LysoPE (18:1)	5.00E-03	0.693a	4.00E-03	0.94	34.24↑	2.68E-02
Cephalin	A13	LysoPE (18:2)	3.00E-03	−0.715a	3.00E-03	0.96	0.23↓	2.55E-03
Cephalin	A14	LysoPE (22:4)	7.00E-03	−0.626a	1.30E-02	0.89	0.41↓	8.50E-03
Cephalin	A17	LysoPE (14:1)	5.00E-03	0.695a	4.00E-03	0.94	11.86↑	2.40E-02
Lecithin	A05	LysoPC (14:0)	2.00E-03	0.713a	3.00E-03	0.98	4.41↑	2.71E-02
Lecithin	A07	LysoPC (15:0)	1.00E-03	0.699a	4.00E-03	0.87	11.32↑	2.48E-02
Lecithin	A08	LysoPC (18:2)	7.00E-03	−0.714a	4.00E-03	0.85	0.30↓	4.09E-03
Lecithin	A09	LysoPC (18:1)	2.50E-02	−0.756a	1.70E-02	0.98	0.19↓	9.85E-04
Lecithin	A10	LysoPC (18:0)	3.00E-03	−0.645a	9.00E-03	0.87	0.44↓	1.24E-02
Lecithin	A11	LysoPC (20:4)	9.00E-03	−0.653a	5.00E-03	0.93	0.24↓	5.24E-03
Bile Acid	A04	Glycocholic acid	5.00E-03	0.723a	2.00E-03	0.94	4.39↑	2.65E-02
Cholic Acid	A12	Cholic acid	1.00E-03	0.736a	2.00E-03	1.00	3.80↑	1.89E-02
Amino Acid	A20	L-Glutamine	3.00E-03	−0.851a	0.00E+00	0.98	0.13↓	3.23E-04
Energy	A19	Glyceric acid	4.00E-03	−0.710a	4.00E-03	0.92	0.33↓	1.06E-02
Energy	A21	Citric acid	2.00E-03	−0.836a	0.00E+00	1	0.45↓	2.31E-03
Fatty Acid	A15	AA	1.00E-03	0.840a	0.00E+00	1.00	2.41↑	3.08E-04
Fatty Acid	A16	DHA	1.00E-03	0.857a	0.00E+00	1.00	2.93↑	3.74E-04
Fatty Acid	A18	HDoHE	7.00E-03	0.547a	3.50E-02	0.93	3.11↑	3.49E-02
Fatty Acid	A22	Myristic acid	2.00E-03	0.821a	0.00E+00	1	4.05↑	6.94E-03
Fatty Acid	A23	Palmitic acid	2.00E-03	0.941a	0.00E+00	1	2.42↑	4.82E-04
Fatty Acid	A24	Eicosadienoic acid	2.00E-03	0.760a	2.00E-03	1	5.07↑	3.14E-02

Ox20THLTB4, 12-Oxo-20-trihydroxy-leukotriene; DHA, Docosahexaenoic acid; HDoHE, Hydroxyl-docosahexaenoic acids; AA, arachidonic acid. a: related to bleeding time.

**Table 2:** Biomarkers associated with MT in uterine tissue.

	No	Metabolites	*P*-value	r	Adjusted *p*-value	AUC	Fold-change (T/M)	FDR
Amino Acid	B01	L-Lysine	3.00E-03	−0.682a	1.00E-02	1.00	0.48↓	0.00E+00
Amino Acid	B04	(R)-3-Hydroxybutyric acid	6.00E-03	0.910b	0.00E+00	0.97	3.28↓	5.20E-03
Amino Acid	B05	Phenylalanine	4.00E-03	0.746b	5.00E-03	1.00	2.12↓	1.65E-02
Amino Acid	B06	L-Glutamine	6.00E-03	−0.639a	2.50E-02	0.98	0.12↑	2.06E-02
Energy	B07	Citric acid	1.60E-02	−0.606a	3.70E-02	0.92	0.49↑	3.18E-02
Nucleotide	B10	Uric acid	4.00E-03	0.649b	2.30E-02	1.00	2.38↓	5.99E-03
Fatty Acid	B02	AA	9.00E-03	0.689b	4.30E-02	0.74	2.31↓	4.08E-02
Fatty Acid	B03	DHA	4.30E-02	0.576a	4.30E-02	0.71	2.11↓	4.95E-02
Fatty Acid	B08	Myristic acid	6.00E-03	0.625a	3.00E-02	0.97	3.53↓	1.76E-02
Fatty Acid	B09	Palmitic acid	4.00E-03	0.816a	1.00E-03	1.00	2.32↓	1.16E-03
Fatty Acid	B11	Stearic acid	4.00E-03	0.850a	0.00E+00	1.00	2.33↓	1.61E-03
Fatty Acid	B12	Eicosadienoic acid	1.00E-02	0.599a	3.90E-02	0.94	2.89↓	3.27E-02

a is related to bleeding time; b is related to blood loss.

### Pathway Analysis

**Figure 5:**
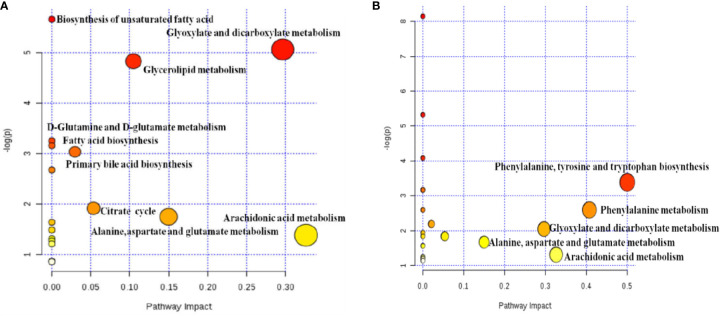
Analysis of metabolic pathway associated with PM **(A)** and MT **(B)** in uterine tissues based on network enrichment and network topology analysis.

Metabolites pathway was analyzed through network enrichment and topology analysis as displayed in **Tables S7** and **S8** and **Figures 5A, B**. There are 18 pathways related to PM and 20 pathways related to MT in uterine tissues. AUB mainly associated with AA metabolism, glyoxylate and dicarboxylate metabolism, alanine, aspartate, and glutamate metabolism, and glycerolipid metabolism. The therapeutic effect of TSD mainly associated with AA metabolism, glyoxylate and dicarboxylate metabolism, and alanine, aspartate, and glutamate metabolism.

## Discussion

According to WHO, there were about 56 million induced abortions between 2010 and 2014. Around 25 million incomplete abortions were estimated worldwide each year mainly in developing countries (Ganatra et al., 2017; Singh et al., 2018). AUB was chiefly initiated by incomplete abortion (Salamonsen et al., 2009) in uterus. TSD is a classic traditional Chinese medicine formula for the treatment of AUB. However, the underlying mechanism is still largely unknown, particularly at the metabolite level. In recent years, metabolomics is developing rapidly in unraveling disease related biochemical events and their underlying mechanisms (Chen et al., 2008). Recent studies have shown that high-throughput omics technologies can be used to discover biomarkers of endometrial-related diseases (Hannan et al., 2010; Díaz-Gimeno et al., 2011). However, the application of metabolomics in the research of abortion was rarely found, mainly in the fields of missed abortion (Wu et al., 2018) and spontaneous abortion (Banerjee et al., 2014). To obtain further insights into the pathogenesis and mechanism, a metabolomic approach was adopted in this study to explore the related biochemical changes of AUB and the treatment of TSD on AUB. Uterus which underwent growth, differentiation, shedding, and regeneration under the combined action of ovarian steroid hormones was used as the sample. Mifepristone and misoprostol-induced incomplete abortion was chosen as the AUB animal model because of its robustness and resemblance to human AUB regarding the degradation and necrosis of decidua and chorion triggered by hormone withdrawal (Evans et al., 2016).

**Figure 6:**
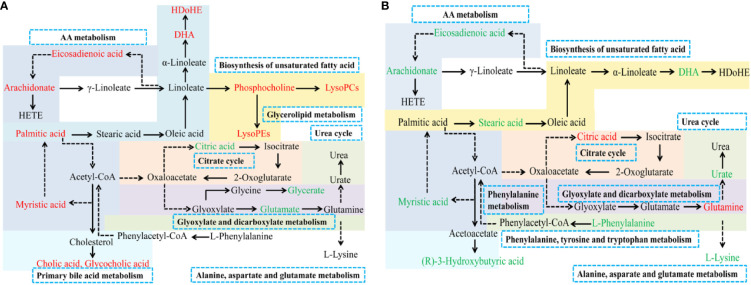
Metabolic pathways associated with PM **(A)** and MT **(B)** in uterine tissue. Red: increased metabolites level; Green: decreased metabolites level; solid arrows: direct metabolic relationship; dashed arrows: indirect metabolic relationship; Blue box: metabolic pathway of metabolites.

To explore the mechanism of TSD on AUB, we analyzed the metabolic profilings of PM and MT. We found that 24 and 12 metabolites related to PM and MT, respectively. The above 24 and 12 biomarkers related to PM and MT mainly fell into 18 and 20 metabolic pathways, respectively. AA metabolism, glyoxylate and dicarboxylate metabolism, alanine, aspartate, and glutamate metabolism were the same metabolic pathways between PM and MT (**Figure 6**).

### TSD Alleviates AUB *via* Amino Acids Signaling

We found that the uric acid in M group showed higher level compared with control, but lower level compared to the treatment. Uric acid is the end-product of purine catabolism (Zieliński et al., 2019). Purine can be synthesized from amino acid, such as glutamine and glycine. Among them, glycine can metabolize into glyceric acid (in glyoxylate and dicarboxylate metabolism pathway), glutamine, alanine, and other amino acids (Alves et al., 2019). The results demonstrated that purine and uric acid metabolism may be disturbed because of AUB.

Meanwhile, the levels of glutamine and glyceric acid which were further metabolites of glycine were lower in M group than control (Alves et al., 2019). AUB may related to the purine metabolism and glyoxylate and dicarboxylate metabolism. TSD may improve the symptoms of AUB *via* improving the above pathways. Interestingly, unlike the uterus, urea not the uric acid had a higher level in the serum in our previous study (Zuo et al., 2019). Alanine which was synthesized from glutamic acid and pyruvate was further metabolized into urea. Therefore, there may be two different pathways of amino acid metabolism: uric acid pathway in the uterus and urea pathway in the blood.

In present study, rats’ uteri in the M group showed lower levels of glutamine, but higher levels in the T group. Glutamine is a non-essential amino acid, which can maintain intestinal barrier on the one hand (Achamrah et al., 2017; Gong et al., 2017; Liu et al., 2018) and promote proliferation and differentiation of intestinal cell on the other (Murnin et al., 2000; Moore et al., 2015). Additionally, glutamine has a biological function as liver protection (Sellmann et al., 2017). It is reported that glutamine was the distinguishing metabolites between spontaneous miscarriage women and control samples (Banerjee et al., 2014). In this study, we found that the levels of glutamine decreased in M group, simultaneously, TSD elevated the level of glutamine. AUB may be related to the disorder of glutamine levels, but the specific mechanisms are not clear. Therapeutic role of TSD on AUB still need further exploration.

### TSD Alleviates AUB *via* Lipids Signaling

Fatty acid, including saturated fatty acid and unsaturated fatty acid, is mainly in the form of ester by the combination with the alcohols. Triglyceride lipase phosphorylates and hydrolyzes into fatty acids through beta oxidation to eventually form acetyl CoA, which further enters into the tricarboxylic acid cycle or synthesizes ketone bodies, including 3-hydroxybutyric acid and acetone. They can also be synthesized under the catalysis of fatty acid synthase system (Rui, 2014). Stearic acid and palmitic acid have been reported to induce the expression of cyclooxygenase 2, which produces prostaglandins and leads to inflammation (Kadotani et al., 2009). Recent studies have shown that too much DHA can lead to the production of free radicals that damage cartilage structure (Izquierdo et al., 2013).

Glycerin phospholipid, mainly including cephalin and lecithin, can be synthesized by fatty acids, phosphoric acid, glycerin, choline, ethanolamine, serine, and inositol. Under the catalysis of phospholipase, lecithin can metabolize to lysophosphatide which holds a wild range of biological effects, such as gastrorrhagia (Neiderhiser and Maksem, 1987), muscle injury (Fuly et al., 2003), induction of inflammation (Hasegawa et al., 2011; Zhao et al., 2016), reactive oxygen species generation (Schilling and Eder, 2010), etc.

In the experiment, we found that the levels of many fatty acids (stearic acid, palmitic acid, myristic acid, et al.) and acetylcarnitine were higher in the M group compared to control, but lower levels in the treatment. In contrast to uterus, the levels of many fatty acids (stearic acid, palmitic acid, et al.) in the blood were lower than control (Zuo et al., 2019). These results may reflect that fatty acids travel through the bloodstream to uterus for utilization under the stress. The levels of cephalin and lecithin are not consistent, respectively.

### TSD Reduces Inflammatory Response *via* AA Signaling

In this study, we found that the level of AA changed dramatically. AA, a major component of the cell membrane lipid content, could be converted into some metabolites, such as prostaglandins (PG), thromboxane (Tx), leukotrienes (LTs), and hydroxyeicosatetraenoic acids (HETEs), which trigger different inflammatory responses (Enyedi et al., 2016; Wang et al., 2019). PG-D_2_ (PGD_2_), derived from AA, binds the PGD_2_ receptor, then stabilizes neutrophils adhesion, shape change, and transmigration to the endothelial cell monolayer. Neutrophils are recruited to a site of tissue inflammation (Tull et al., 2009). Neutrophils are rich sources of defensins and whey acid protein motif proteins, which play important roles in ensuring microbial protection while the epithelial barrier is disrupted. An extracellular vesicle produced by a neutrophil, allows shuttling of AA into platelets and then synthesizes Tx A_2_ (TxA_2_) (Rossaint et al., 2016). These compounds derived from AA can not only act as mediators themselves but can also modulate other processes, such as platelet aggregation, coagulation, smooth muscle contraction, leukocyte chemotaxis, inflammatory cytokine production, and immune function (Kudo and Murakami, 2002; Calder, 2003; Zhao and Imig, 2003; Pompeia et al., 2003).

TSD, a TCM formula, had an effect of anti-inflammation (Wu et al., 2011), as its ingredients attenuate inflammatory response, such as hydroxysafflor yellow A (Pei et al., 2017), ferulic acid (Yuan et al., 2016; Kwon et al., 2019), amygdalin (Hwang et al., 2008), ligustilide (Or et al., 2011; Kuang et al., 2014), verbascoside (Lai et al., 2019). This was consistent with histological results. In drug treatment group, the quantities of neutrophils observed were less than that of medical aborting group (Zhang et al., 2018). We speculated that the AUB was accompanied by an inflammatory response which may be mediated by AA. TSD reduced the inflammatory responses through the down regulation of the level of AA (Ma et al., 2018). This is consistent with the result of metabolomics where the level of AA increased in PM but reduced in MT significantly.

TSD is mainly used to treat blood stasis syndrome (AUB or dysmenorrhea) in gynecology, however, the mechanism of therapeutic effect has not been fully explained. In this study, the treatment of AUB by TSD was reported from the perspective of metabolic regulation based on UFLC-IT-TOF/MS and GC/MS metabolomics. There are also some limitations to this study. The biomarkers obtained from the analysis of LC/MS and GC/MS mainly concerned with amino acids or lipids, but lacks a large amount of glucose involved in energy metabolism. Whether there are false positive for these biomarkers requires further verification. Additionally, the data processing and analysis software used in metabolomics also has some subtle differences.

## Conclusion

In conclusion, adopting untargeted GC/MS and LC/MS spectroscopy combined with multivariate statistical analysis, we analyzed the uterine metabolic profiling of TSD on AUB. The metabolic profiles of AUB are mainly related to AA metabolism, glyoxylate and dicarboxylate metabolism, alanine, aspartate, glutamate metabolism, and glycerolipid metabolism. TSD has a significant regulatory effect on multiple biomarkers of AUB mainly through AA metabolism, glyoxylate and dicarboxylate metabolism, alanine, aspartate, glutamate metabolism. This is the first uterine metabolomics study focusing on AUB and provide a new idea for explaining the mechanism of TSD on AUB. Certainly, functional studies and clinical specimen analysis will be needed to further demonstrate the therapeutic mechanism of TSD on AUB. The association of palmitic acid, stearic acid, and uric acid with the AUB provides a knowledge-based hypothesis for further research.

## Data Availability Statement

The original contributions presented in the study are included in the article/supplementary material, further inquiries can be directed to the corresponding authors.

## Ethics Statement

The animal study was reviewed and approved by the Ethical Committee of Anhui Medical University.

## Author Contributions

YZ and CZ performed the majority of the experiment and analyzed the data. YZ drafted and revised the manuscript. JW, XL, and LH supported several experiments. DP, WC, SG, and CP supervised the research and revised the manuscript.

## Funding

This work was supported by the National Natural Science Foundation of China (81473387, 81503291, 81703805, 81804043), Anhui Province Key Laboratory of Chinese Medicinal Formula (2019AKLCMF02).

## Conflict of Interest

The authors declare that the research was conducted in the absence of any commercial or financial relationships that could be construed as a potential conflict of interest.
