# Supplementary material for: Uterine Metabolomics Reveals Protection of Taohong Siwu Decoction Against Abnormal Uterine Bleeding
Source: Front Pharmacol. 2020 Sep 11;11:507113. doi: 10.3389/fphar.2020.507113 (PMC7518030; doi:10.3389/fphar.2020.507113)
Supplement: Supplementary file 1 [file DataSheet_1.pdf]

Supplementary Material

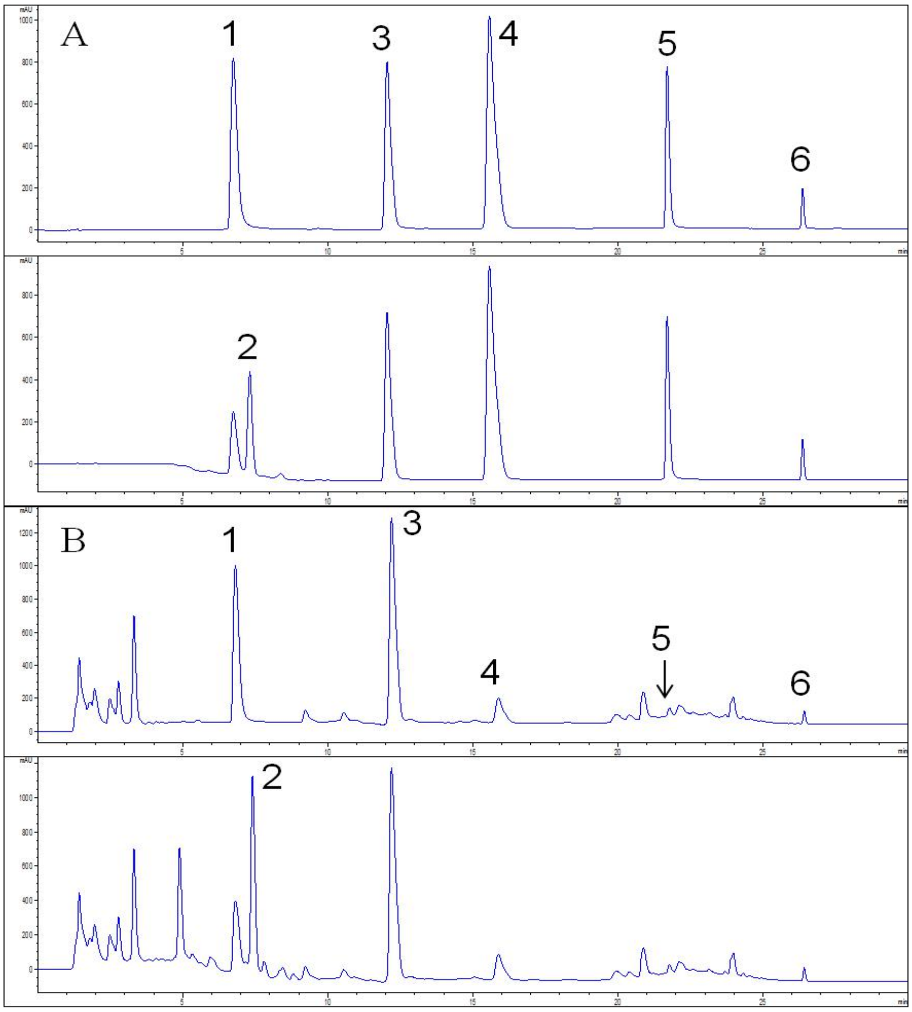

Fig. S1　UHPLC chromatogram of TSD indicator component (A: Control, B: TSD; 1: Hydroxysafflor yellow A; 2: Amygdalin; 3: Paeoniflorin; 4: Ferulic Acid; 5: Verbascoside; 6: Ligustilide)

Tbl. S1　Content of TSD indicator components ($\bar{x} \pm s$, n=6)

chemical components	t_R (min)	Concentration (μg/mL)
Hydroxysafflor yellow A	6.742	253.3±5.54
Amygdalin	7.312	506.8±5.69
Paeoniflorin	12.046	665.9±6.39
Ferulic Acid	15.574	35.94±0.49
Verbascoside	21.701	13.51±3.42
Ligustilide	26.370	265.2±0.94

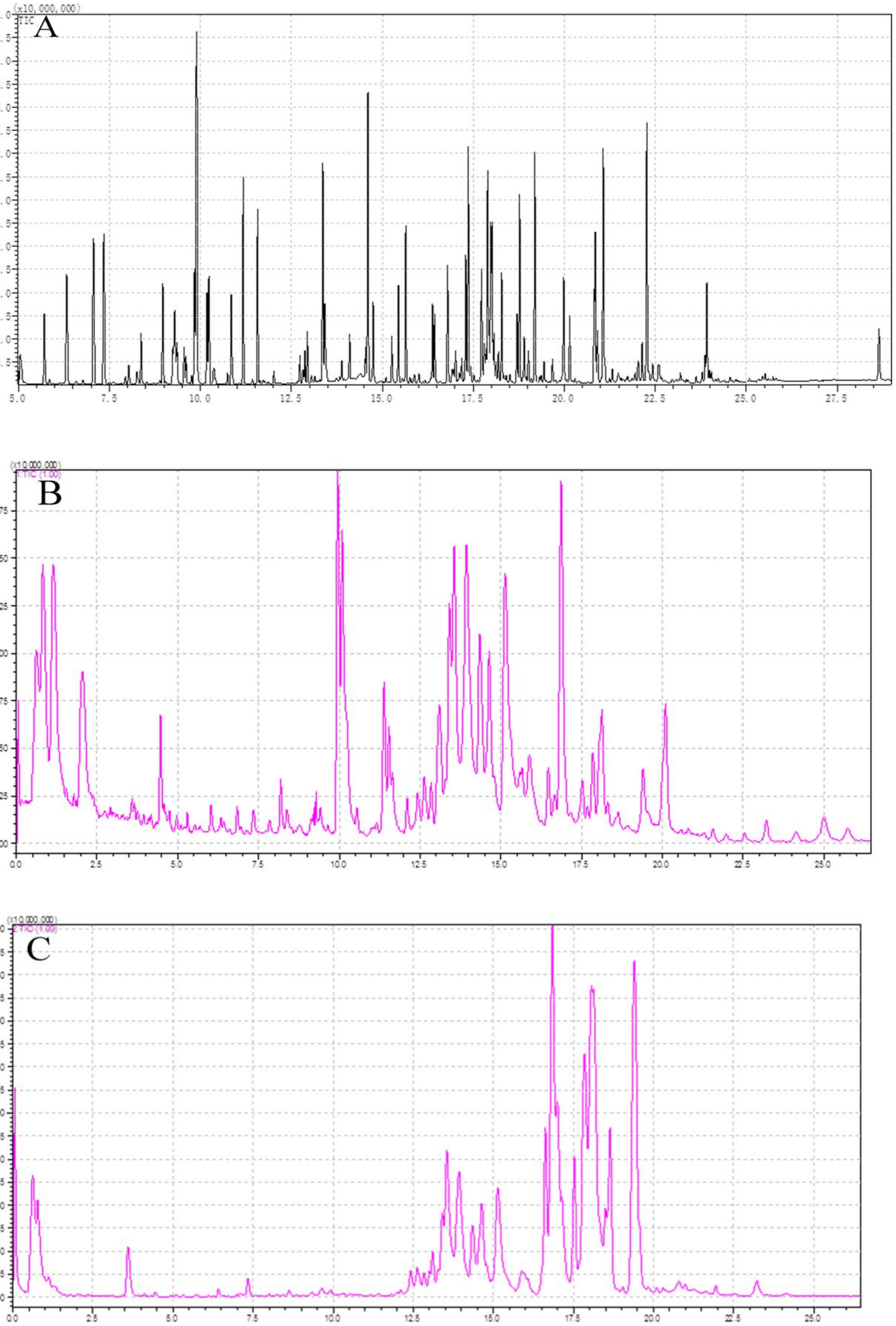

Fig. S2A-C Typical TICs of uterine tissues generated by GC/MS (A), LC/MS(ESI+) (B), LC/MS(ESI-) (C)

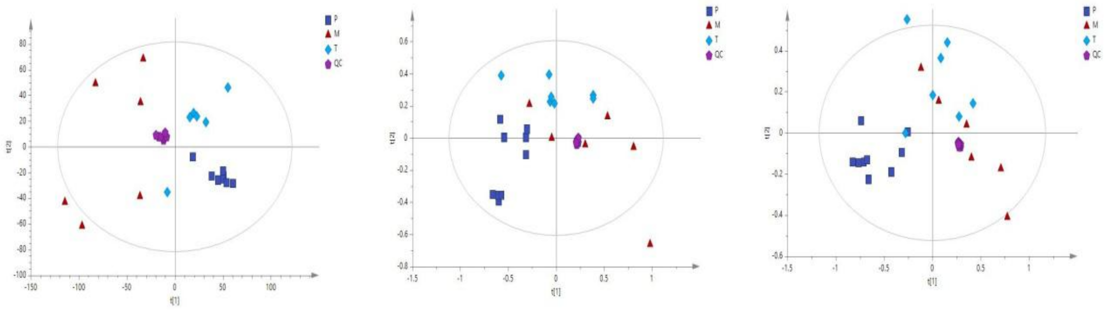

A B C

Fig. S3 Scores plots of PCA for P, M, T and QC samples in uterine tissues GC/MS (A) and UFLC-IT-TOF/MS (B: ESI +; C: ESI -)

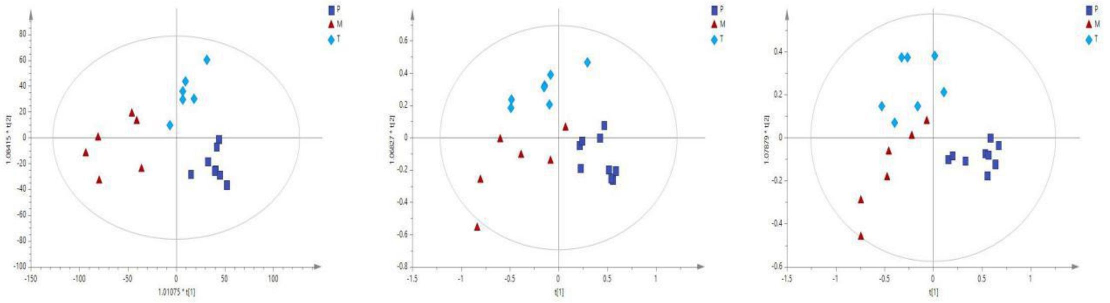

A B C

Fig. S4 Scores plots of OPLS-DA for P, M, T and QC samples in uterine tissues GC/MS (A) and UFLC-IT-TOF/MS (B: ESI +; C: ESI -)

Tbl. S2 Results of the cross validation, permutation tests among P, M and T in uterine tissues

**Model**			**R^2X**	**R^2Y**	**Q^2**	**R2 inter**	**Q2 inter**	***P*-value (CV-ANOVA)**
PCA		GC/MS	0.671	/	0.412	/	/	/
PCA		LC/MS(ESI+)	0.722	/	0.463	/	/	/
PCA		LC/MS(ESI-)	0.734	/	0.504	/	/	/
OPLS-DA	PMT	GC/MS	0.458	0.802	0.639	/	/	/
OPLS-DA	PMT	LC/MS(ESI+)	0.528	0.695	0.592	/	/	/
OPLS-DA	PMT	LC/MS(ESI-)	0.57	0.729	0.596	/	/	/
OPLS-DA	P vs M	GC/MS	0.523	0.966	0.889	0.647	-0.249	2.55E-04
OPLS-DA	P vs M	LC/MS(ESI+)	0.555	0.892	0.683	0.687	-0.036	1.42E-02
OPLS-DA	P vs M	LC/MS(ESI-)	0.612	0.957	0.848	0.206	-0.233	4.28E-04
OPLS-DA	M vs T	GC/MS	0.529	0.944	0.771	0.805	-0.023	2.12E-02
OPLS-DA	M vs T	LC/MS(ESI+)	0.562	0.894	0.597	0.41	-0.089	4.90E-02
OPLS-DA	M vs T	LC/MS(ESI-)	0.498	0.881	0.475	0.693	-0.025	2.21E-02

Tbl. S3 The tentatively identified biomarkers by GC/MS related to PM

No	Biomarker	Ion m/z	Ion RT	Similar
1	L-Alanine	116.1515	7.074	94
2	Urea	147.11	9.296	93
3	L-Proline	142.1664	10.246	95
4	Glyceric acid	119.05	10.751	94
5	Pyrimidine	120.0731	10.857	92
6	Serine	45.05	11.183	96
7	L-Threonine	117.103	11.58	95
8	L-Aspartic acid	61.041	13.361	93
9	Glutamic acid	69.0705	14.588	95
10	L-Lysine	271.1444	15.653	87
11	Phosphoric acid	445.1625	16.384	95
12	L-Glutamine	129.1146	16.448	85
13	Citric acid	257.1045	17.02	85
14	Myristic acid	117.0661	17.15	85
15	L-Tyrosine	190.0784	18.289	90
16	Palmitic acid	313.2993	19.199	93
17	Uric acid	111.0734	19.996	85
18	Heptadecanoic acid	167.1602	20.156	87
19	Linoleic acid	234.2336	20.827	94
20	Stearic acid	54.1022	21.077	95
21	D-Fructose	77.0431	21.476	89
22	Eicosadienoic acid	109.1194	22.595	85
23	Hexadecanoic acid	89.0733	24.062	84
24	Octadecanoic acid	95.1369	25.525	91

Tbl. S4 The tentatively identified biomarkers by LC/MS related to PM

No	Metabolites	ion forms	ESI (+/-)	Ion m/z	Ion RT	P-value	VIP	MS/MS fragment or Similarity
1	L-Phenylalanine	[M+H]+	+	166.0853	1.152	0.013	3.8728	120.0797, 149.0548
2	L-Tyrosine	[M+H]+	+	182.0804	0.805	0.013	2.137	165.0544;136.0707
3	L-Acetylcarnitine	[M+H]+	+	204.122	0.768	0.002	2.0701	
4	PE(P-16:0e/0:0)	[M+H]+	+	438.2972	13.559	0.005	7.0623	420.2866, 284.2937, 266.2840,
	PE(P-16:0e/0:0)	[M-H]-	-	436.2669	13.55	0.005	5.2791	375.2149, 196.0328
5	LysoPE(16:0)	[M+H]+	+	454.2915	13.098	0.034	2.3533	436.2792, 313.2705
	LysoPE(16:0)	[M-H]-	-	452.2616	13.091	0.025	3.0161	255.2240, 214.0468
6	Glycocholic acid	[M+H]+	+	466.3269	15.151	0.005	5.8558	448.3151, 312.3238,294.3187
	Glycocholic acid	[M-H]-	-	464.2963	15.14	0.005	4.5248	196.0350, 267.2600
7	LysoPC(14:0)	[M+H]+	+	468.3381	15.145	0.002	1.7103	184.0720, 450.2949, 468.2967
8	LysoPE(18:1)	[M+H]+	+	480.3412	12.715	0.002	1.5192	462.296, 389.1993, 462.296, 339.2897
	LysoPE(18:1)	fragment	+	462.296	13.007	0.005	3.0012	389.1993, 339.2897
	LysoPE(18:1)	[M-H]-	-	478.2752	13.485	0.013	2.5946	281.2398
9	LysoPC(15:0)	[M+H]+	+	482.3577	13.934	0.001	5.9909	464.3114, 240.098, 181.024, 184.0774
	LysoPC(15:0)	[M+HCOO]-	-	526.3297	13.933	0.001	3.0908	466.3119
10	LysoPC(16:0)	[M+H]+	+	496.3377	13.403	0.018	4.081	184.3297, 478.3273
	LysoPC(16:0)	[M+HCOO]-	-	540.3088	13.397	0.001	4.181	480.2900;
	LysoPC(16:0)	fragment	-	480.2914	13.396	0.001	1.6432	255.2237, 480.2898
11	LysoPC(18:2)	[M+H]+	+	520.3371	12.838	0.007	2.1492	502.3278, 184.0731
	LysoPC(18:2)	[M+HCOO]-	-	564.3083	12.833	0.005	1.8143	504.2882, 279.2219
12	LysoPC(18:1)	[M+H]+	+	522.3535	13.814	0.025	2.94	184.0736, 504.3425, 523.3550
	LysoPC(18:1)	[M+HCOO]-	-	566.3232	13.526	0.002	1.3989	506.3038, 281.2343
13	LysoPC(18:0)	[M+H]+	+	524.3681	15.1	0.018	4.0443	506.5580, 184.0731
	LysoPC(18:0)	[M+HCOO]-	-	568.339	14.791	0.013	1.7463	508.3179, 283.2554, 224.0639
	LysoPC(18:0)	fragment	-	508.3206	15.089	0.003	1.5232	508.3179, 283.2554, 224.0639
14	LysoPC(20:4)	[M+H]+	+	544.3369	12.847	0.003	2.8729	526.3264, 184.0724
	LysoPC(20:4)	[M+HCOO]-	-	588.3068	12.841	0.005	2.3819	303.2199, 259.2352, 528.2851
15	LysoPC(20:3)	[M+H]+	+	546.2808	12.368	0.045	1.2108	528.2856, 361.2719
16	Ox20THLTB4	[M-H]-	-	381.1604	18.15	0.001	4.6262	363.1496, 345.3188, 319.1611
17	Cholic acid	[M-H]-	-	407.1734	18.492	0.001	2.9418	389.1648, 371.1539
18	Taurocholic acid	fragment	-	462.2804	13.931	0.018	2.7999	401.2312, 265.2451,196.350
19	LysoPE 18:2	[M-H]-	-	476.2595	12.58	0.003	1.3124	279.2228
20	LysoPE(22:4)	[M+H]+	+	530.3221	13.746	0.013	1.1421	389.2994, 512.3166
	LysoPE(22:4)	[M-H]-	-	528.2885	13.74	0.007	1.3923	331.2550, 287.2667
21	AA	[M-H]-	-	303.2218	16.837	0.001	7.1442	259.5434, 285.2095
22	DHA	[M-H]-	-	305.2285	16.835	0.001	1.3644	283.2286, 191.1777
23	LysoPE(14:1)	[M-H]-	-	422.2519	12.783	0.005	1.1187	196.0365, 225.2069, 361.1991
24	HDoHE	[M-H]-	-	343.2153	13.753	0.007	1.084	281.2416

Note: Ox20THLTB4:12-Oxo-20-trihydroxy-leukotriene; DHA: Docosahexaenoic acid

HDoHE: Hydroxyl-docosahexaenoic acids; AA: arachidonic acid

Tbl. S5 The tentatively identified biomarkers by GC/MS related to MT

No	Compound	Ion m/z	Ion RT	Similar
1	Lactic acid	51.0432	6.338	96
2	L-Alanine	116.1515	7.074	94
3	Glycine	102.1093	7.355	95
4	(R)-3-Hydroxybutyric acid	117.0986	8.036	96
5	L-Valine	88.026	8.971	98
6	Urea	135.0741	9.299	93
7	Leucine	53.0209	9.849	95
8	L-Isoleucine	246.1302	10.19	84
9	Serine	69.0517	11.182	96
10	L-Threonine	58.0522	11.58	95
11	L-Aspartic acid	60.0492	13.369	93
12	L-Proline	69.0492	13.429	96
13	Butanoic acid	155.1582	13.473	88
14	Glutamic acid	185.0652	14.609	95
15	Phenylalanine	150.1217	14.75	94
16	L-Asparagine	145.1098	15.264	93
17	L-Lysine	116.0978	15.644	87
18	Phosphoric acid	258.1059	16.381	95
19	L-Glutamine	129.1146	16.448	85
20	Ornithine	299.117	16.958	93
21	Citric acid	222.0795	17.024	85
22	Myristic acid	201.1042	17.147	85
23	L-Tyrosine	366.1643	18.282	90
24	Palmitic acid	313.2993	19.199	93
25	Uric acid	60.063	19.992	85
26	Heptadecanoic acid	139.1659	20.155	87
27	Linoleic acid	212.1049	20.836	94
28	Stearic acid	286.2467	21.075	95
29	D-Fructose	77.0431	21.476	89
30	Eicosadienoic acid	152.1787	22.603	85
31	Hexadecanoic acid	70.0862	24.018	84
32	Octadecanoic acid	401.3491	25.522	91

Tbl. S6 The tentatively identified biomarkers by LC/MS related to MT

No	Metabolites	ion forms	ESI (+/-)	Ion m/z	Ion RT	P-value	VIP	MS/MS fragment or Similarity
1	L-Lysine	[M+H]+	+	146.1637	0.529	0.003	1.4564	130.086
2	L-Tryptophan	[M+H]+	+	205.0953	2.049	0.046	3.3167	188.0688, 146.0671
3	arachidonic acid	[M-H]-	-	303.2218	16.837	0.015	5.1170	259.5434, 285.2095
4	DHA	[M-H]-	-	305.2285	16.835	0.020	1.0158	283.2286, 191.1777
5	LysoPC(16:0)	[M+H]+	+	496.3377	13.403	0.046	5.4333	184.3297, 478.3273
	LysoPC(16:0)	[M+HCOO]-	-	540.3088	13.397	0.032	5.0007	480.2900;
6	LysoPC(18:0)	[M+H]+	+	524.3681	15.1	0.022	5.7643	506.5580, 184.0731
	LysoPC(18:0)	[M+HCOO]-	-	568.339	14.791	0.015	3.1665	508.3179, 283.2554, 224.0639
7	Taurocholic acid	fragment	-	462.2804	13.931	0.032	3.5134	401.2312, 265.2451,196.350

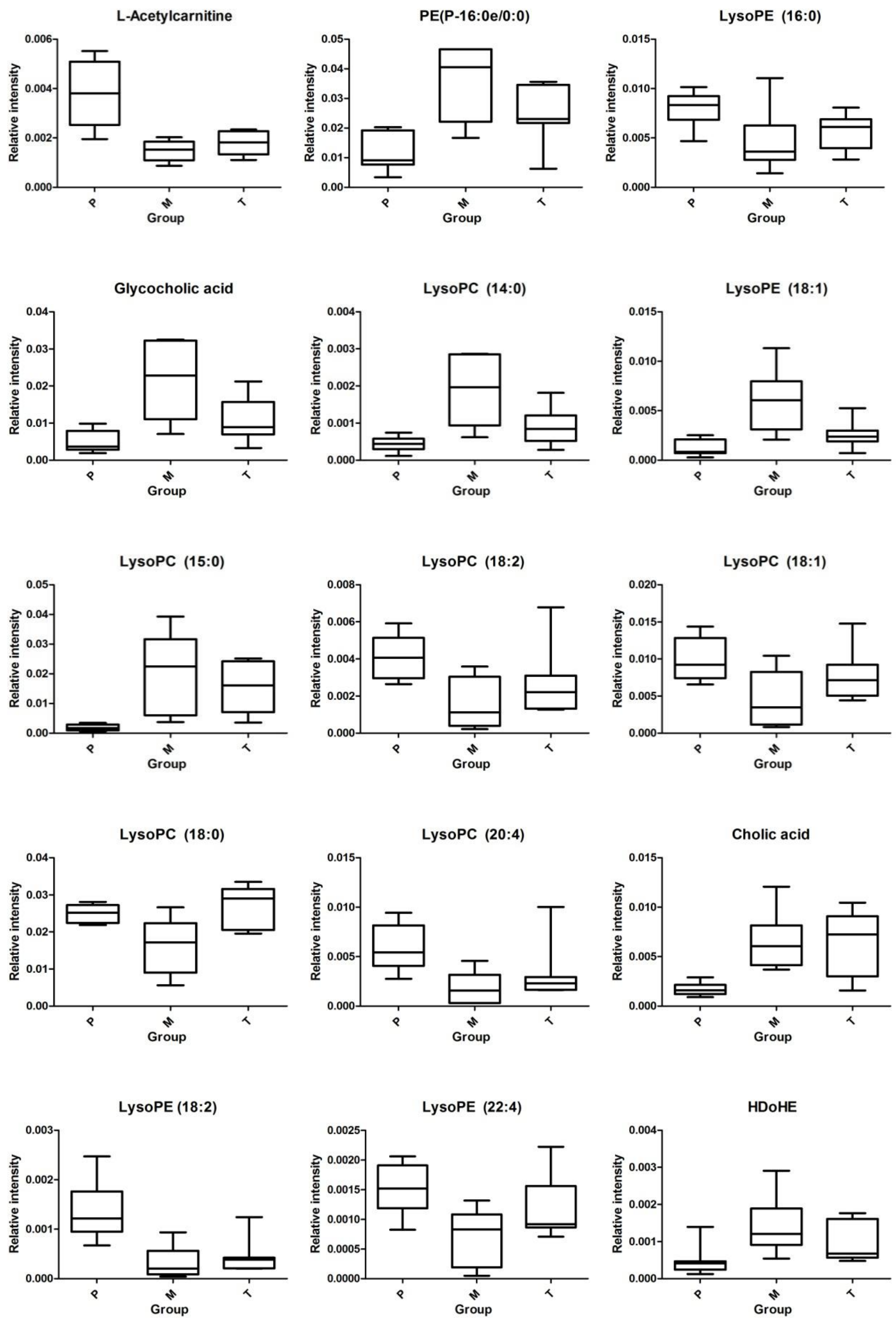
L-Acetylcarnitine
PE(P-16:0e/0:0)
LysoPE (16:0)
Glycocholic acid
LysoPC (14:0)
LysoPE (18:1)
LysoPC (15:0)
LysoPC (18:2)
LysoPC (18:1)
LysoPC (18:0)
LysoPC (20:4)
Cholic acid
LysoPE (18:2)
LysoPE (22:4)
HDoHE
Relative intensity
Group
P
M
T

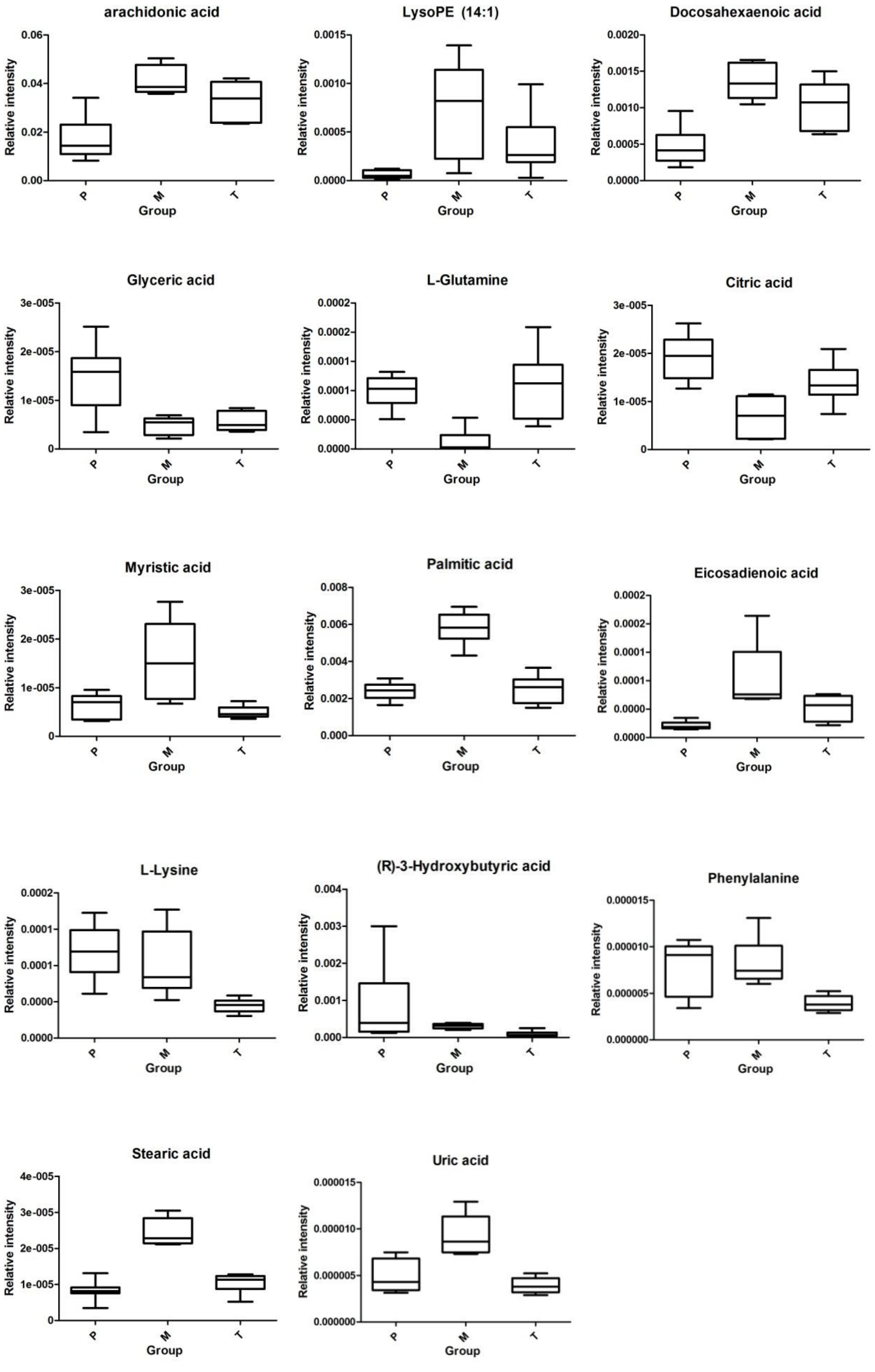

Fig. S5 Changes of the contents of biomarkers in uterine tissue

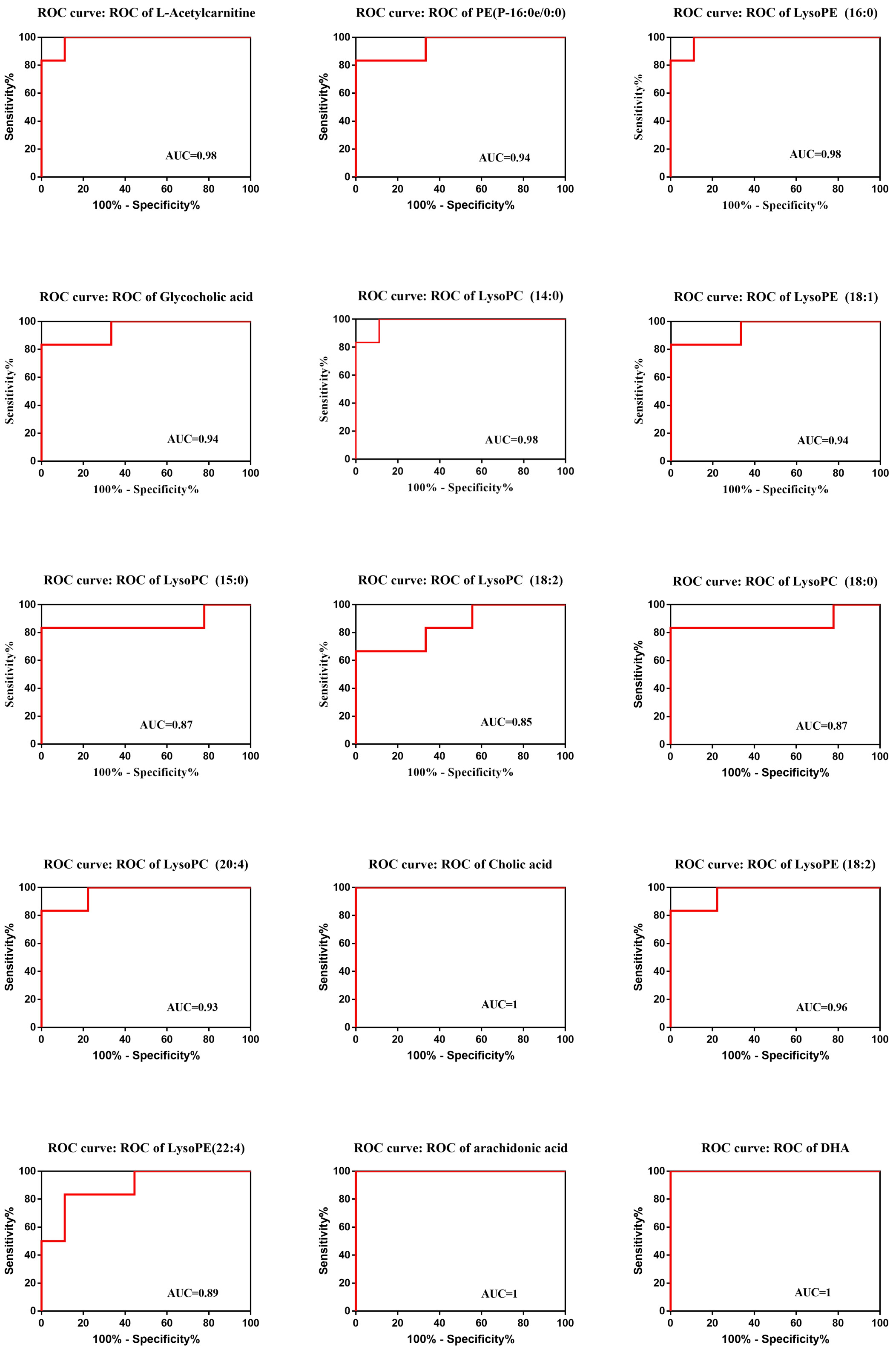
ROC curve: ROC of L-Acetylcarnitine
AUC=0.98
ROC curve: ROC of PE(P-16:0e/0:0)
AUC=0.94
ROC curve: ROC of LysoPE (16:0)
AUC=0.98
ROC curve: ROC of Glycocholic acid
AUC=0.94
ROC curve: ROC of LysoPC (14:0)
AUC=0.98
ROC curve: ROC of LysoPE (18:1)
AUC=0.94
ROC curve: ROC of LysoPC (15:0)
AUC=0.87
ROC curve: ROC of LysoPC (18:2)
AUC=0.85
ROC curve: ROC of LysoPC (18:0)
AUC=0.87
ROC curve: ROC of LysoPC (20:4)
AUC=0.93
ROC curve: ROC of Cholic acid
AUC=1
ROC curve: ROC of LysoPE (18:2)
AUC=0.96
ROC curve: ROC of LysoPE(22:4)
AUC=0.89
ROC curve: ROC of arachidonic acid
AUC=1
ROC curve: ROC of DHA
AUC=1
Sensitivity%
100% - Specificity%

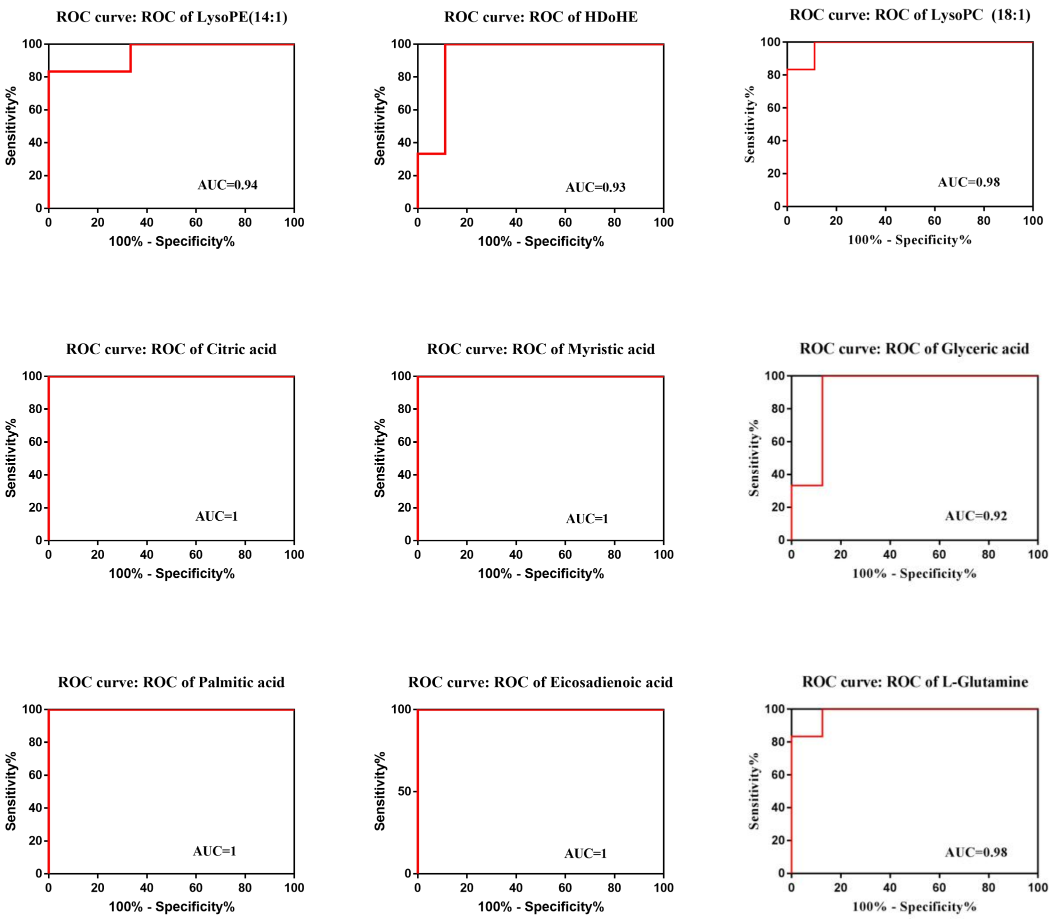

Fig. S6 Biomarker ROC curves associated with PM in uterine tissue

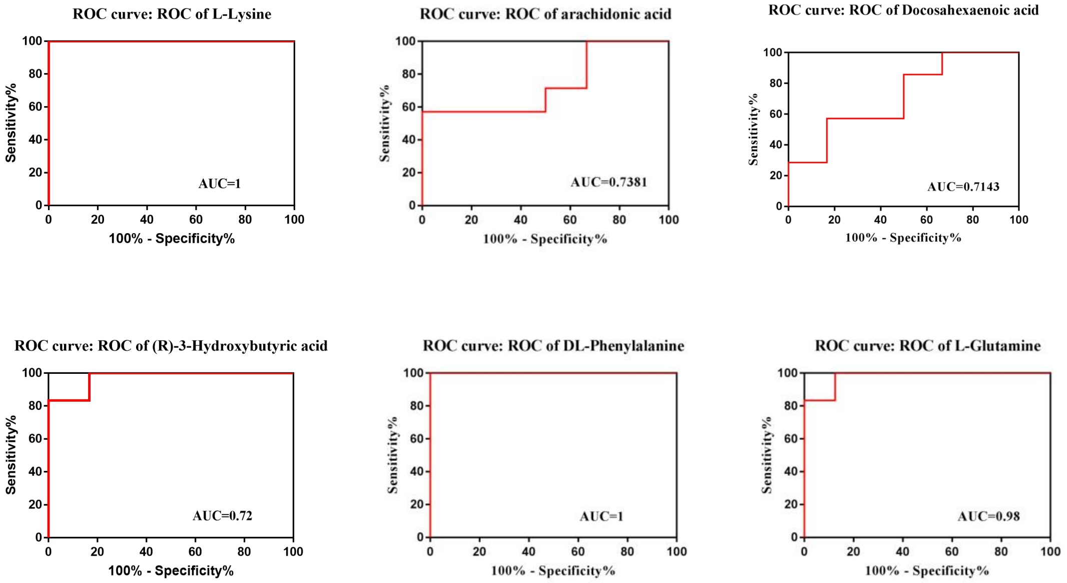

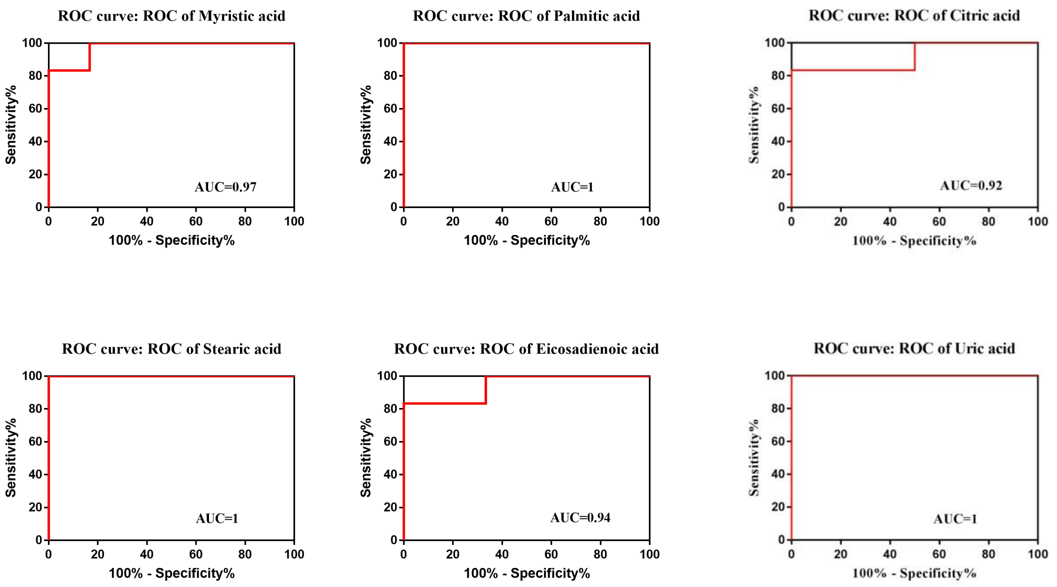

Fig. S7 Biomarker ROC curves associated with MT in uterine tissue

Tbl. S7 Metabolic pathway analysis of biomarkers associated with PM in uterine tissues

No	Pathway name	Total	Hits	-log(p)	Impact
PM1	Arachidonic acid metabolism	36	1	1.3129	0.32601
PM2	Glyoxylate and dicarboxylate metabolism	16	2	4.8871	0.2963
PM3	Alanine, aspartate and glutamate metabolism	24	1	1.6723	0.14979
PM4	Glycerolipid metabolism	18	1	1.9368	0.10471
PM5	Citrate cycle (TCA cycle)	20	1	1.8392	0.05356
PM6	Glycerophospholipid metabolism	30	1	1.4722	0.04444
PM7	Primary bile acid biosynthesis	46	2	2.8753	0.02976
PM8	Biosynthesis of unsaturated fatty acids	42	4	8.1458	0
PM9	D-Glutamine and D-glutamate metabolism	5	1	3.167	0
PM10	Fatty acid biosynthesis	43	2	2.9974	0
PM11	Nitrogen metabolism	9	1	2.5949	0
PM12	Fatty acid elongation in mitochondria	27	1	1.5661	0
PM13	Glycine, serine and threonine metabolism	32	1	1.4154	0
PM14	Fatty acid metabolism	39	1	1.2443	0
PM15	Pyrimidine metabolism	41	1	1.2018	0
PM16	Arginine and proline metabolism	44	1	1.1426	0
PM17	Aminoacyl-tRNA biosynthesis	67	1	0.80817	0
PM18	Purine metabolism	68	1	0.79705	0

Tbl. S8 Metabolic pathway analysis of biomarkers associated with MT in uterine tissues

No	Pathway name	Total	Hits	-log(p)	Impact
MT1	Phenylalanine, tyrosine and tryptophan biosynthesis	4	1	3.3862	0.5
MT2	Phenylalanine metabolism	9	1	2.5949	0.40741
MT3	Arachidonic acid metabolism	36	1	1.3129	0.32601
MT4	Glyoxylate and dicarboxylate metabolism	16	1	2.0468	0.2963
MT5	Alanine, aspartate and glutamate metabolism	24	1	1.6723	0.14979
MT6	Citrate cycle (TCA cycle)	20	1	1.8392	0.05356
MT7	Purine metabolism	68	2	2.1908	0.02077
MT8	Biosynthesis of unsaturated fatty acids	42	4	8.1458	0
MT9	Fatty acid biosynthesis	43	3	5.3231	0
MT10	Aminoacyl-tRNA biosynthesis	67	3	4.0843	0
MT11	Synthesis and degradation of ketone bodies	5	1	3.167	0
MT12	D-Glutamine and D-glutamate metabolism	5	1	3.167	0
MT13	Biotin metabolism	5	1	3.167	0
MT14	Nitrogen metabolism	9	1	2.5949	0
MT15	Glycerolipid metabolism	18	1	1.9368	0
MT16	Butanoate metabolism	20	1	1.8392	0
MT17	Fatty acid elongation in mitochondria	27	1	1.5661	0
MT18	Fatty acid metabolism	39	1	1.2443	0
MT19	Pyrimidine metabolism	41	1	1.2018	0
MT20	Arginine and proline metabolism	44	1	1.1426	0
